# Neural Turing Machines for efficient natural language summarization: architecture, optimization, and performance analysis

**DOI:** 10.3389/frai.2026.1889866

**Published:** 2026-08-31

**Authors:** Kartik Reddy Katti, Kartikeya Reddy Katti, Amanul Islam

**Affiliations:** 1 Nocancer.ai, San Francisco, CA, United States; 2 Department of Computer Science, University of Colorado, Colorado Springs, CO, United States

**Keywords:** computational efficiency, deep learning, external memory, natural language processing, Neural Turing Machines, text summarization

## Abstract

**Introduction:**

Abstractive text summarization remains a fundamental challenge in Natural Language Processing (NLP), particularly for long documents that require models to preserve long-range dependencies and maintain semantic coherence. Although Transformer-based architectures have achieved strong summarization performance, their full self-attention mechanism scales quadratically with sequence length and often requires input truncation in long-context applications.

**Methods:**

This study presents a Neural Turing Machine (NTM)-based framework for abstractive text summarization. The proposed architecture combines a two-layer Bidirectional Long Short-Term Memory (BiLSTM) controller with an addressable external memory bank. Differentiable read and write operations decouple contextual storage from recurrent computation, enabling the persistent retrieval of salient information across extended input sequences. Detailed preprocessing, implementation, training, decoding, and evaluation settings are provided to support reproducibility. The framework was evaluated on the CNN/Daily Mail benchmark and compared with LSTM, Transformer, and BART baselines, as well as published high-performing systems, including PEGASUS, SimCLS, and BRIO. Ablation studies, learning-rate sensitivity analysis, long-context evaluation, inference-time scaling, statistical testing, qualitative error analysis, and memory-access visualization were also conducted.

**Results:**

The proposed NTM model achieved ROUGE-1, ROUGE-2, ROUGE-L, and BLEU scores of 47.8, 23.5, 44.6, and 20.1, respectively. Under the controlled experimental protocol, it outperformed the evaluated LSTM, Transformer, and BART baselines. Comparisons with published results indicate that the model is competitive with recent high-performing summarization systems. The additional analyses demonstrate that the external memory mechanism improves contextual retention and summarization stability, particularly for longer input sequences, while exhibiting favorable inference-time scaling behavior.

**Discussion:**

These findings demonstrate that integrating an addressable external memory with a BiLSTM controller offers an effective approach to abstractive summarization, particularly when processing long documents. The proposed framework provides competitive summarization performance while reducing dependence on computationally expensive full self-attention. The results highlight the potential of external-memory architectures as a scalable and stable alternative for long-context text summarization.

## 1 Introduction

The rapid proliferation of digital text has necessitated the development of automated systems capable of distilling vast amounts of information into concise and meaningful summaries. Text summarization, categorized into extractive and abstractive methods, has seen significant advancements with the rise of deep learning. Abstractive summarization, in particular, aims to generate new sentences that capture the essence of the source text, a task that requires a deep understanding of semantics and long-range context.

Early sequence-to-sequence (Seq2Seq) models utilizing Recurrent Neural Networks (RNNs) and Long Short-Term Memory (LSTM) networks struggled with the “vanishing gradient” problem, which limited their ability to capture dependencies beyond a few dozen tokens. The introduction of the attention mechanism and subsequently the Transformer architecture revolutionized the field by allowing models to attend to any part of the input sequence regardless of distance. However, the self-attention mechanism's *O*(*n*^2^) complexity makes it computationally prohibitive for very long documents, such as legal contracts or academic papers.

Neural Turing Machines (NTMs), introduced by (Graves et al. 2014), offer a fundamentally different approach. By integrating a differentiable memory bank with a neural controller, NTMs can perform read and write operations that are learned through backpropagation. This architecture mimics the von Neumann architecture of traditional computers, providing a way to store and retrieve information over long intervals without the constraints of a fixed hidden state size.

In this work, we introduce a novel Neural Turing Machine (NTM)-based summarization architecture with a bidirectional LSTM (BiLSTM) controller and differentiable read/write heads for an external memory. The proposed framework is specifically designed to address the challenges of long-range dependency modeling in abstractive text summarization. The key contributions of this work are summarized as follows:

- We propose an NTM-based framework for abstractive summarization that integrates a two-layer BiLSTM controller with a fixed-size differentiable memory matrix. The controller generates content-based and temporal memory-addressing parameters, enabling explicit storage and retrieval of document-level context during summary generation.
- We provide a reproducible implementation protocol, including dataset split, pre-processing rules, vocabulary construction, truncation strategy, controller dimensions, memory size, optimizer configuration, gradient clipping, teacher forcing, beam-search decoding, random seeds, and metric computation. These details are added to make the proposed framework scientifically assessable and easier to replicate.
- We evaluate the framework using controlled baselines implemented under the same experimental protocol and contextualize the results against published strong summarization systems such as PEGASUS, SimCLS, BRIO, and reranking-based summarization models. This distinction avoids overclaiming and clarifies whether comparisons are directly controlled or based on published reference results.
- We conduct ablation, efficiency, long-context, sensitivity, statistical, and qualitative analyses to explain not only whether the model performs well, but also why the external memory improves contextual retention and where the method remains limited.

The remainder of this paper is organized as follows. Section 2 reviews the related work in text summarization and memory-augmented neural networks. Section 3 details the proposed NTM architecture and its implementation for summarization. Section 4 describes the experimental setup, including the dataset and training procedures. Section 5 presents the experimental results, including performance metrics, ablation studies, and efficiency analysis. Section 6 provides a discussion of the findings, and Section 7 concludes the paper with suggestions for future research.

## 2 Related work

### 2.1 Abstractive text summarization

Abstractive text summarization has evolved significantly with the advancement of deep learning and neural sequence modeling techniques. Early approaches primarily relied on sequence-to-sequence (Seq2Seq) architectures using recurrent neural networks and Long Short-Term Memory (LSTM) units for text generation (Sutskever et al., 2014). (Rush et al. 2015) introduced one of the earliest neural abstractive summarization systems using an attention-based encoder-decoder framework. The introduction of attention mechanisms further improved contextual learning and alignment between source and target sequences (Bahdanau et al., 2015; Luong et al., 2015).

The emergence of Transformer architectures (Vaswani et al., 2017) significantly improved summarization performance by enabling parallel processing and better contextual representation through self-attention mechanisms. Several large-scale pre-trained language models, including BERT (Devlin et al., 2019), GPT (Radford et al., 2018), BART (Lewis et al., 2020), PEGASUS (Zhang et al., 2020), and T5 (Raffel et al., 2020), achieved state-of-the-art results on benchmark summarization datasets through transfer learning and large-scale pre-training. Pointer-generator networks (See et al., 2017) and reinforcement learning-based summarization models (Paulus et al., 2018) were also proposed to reduce repetition and improve factual consistency in generated summaries. Despite their strong performance, these models are generally limited by fixed input lengths and computational complexity when processing long documents.

### 2.2 Handling long sequences

Handling long-context documents remains a major challenge in abstractive summarization systems. Standard Transformer models suffer from quadratic computational complexity due to pairwise self-attention operations (Vaswani et al., 2017). To address this limitation, several efficient Transformer variants have been proposed. (Beltagy et al. 2020) introduced sparse sliding-window attention to improve scalability for long documents, while (Zaheer et al. 2020) utilized sparse global attention mechanisms to reduce memory consumption. (Kitaev et al. 2020) employed locality-sensitive hashing to optimize attention computation, and (Wang et al. 2020) reduced self-attention complexity through low-rank matrix approximations. (Choromanski et al. 2021) further improved efficiency by approximating softmax attention with kernel-based methods.

In addition to efficient attention mechanisms, discourse-aware and hierarchical summarization approaches have also been explored for long-document understanding. (Cohan et al. 2018) proposed a discourse-aware attention model capable of capturing hierarchical document structures in scientific papers. (Dai et al. 2019) extended contextual retention using segment-level recurrence mechanisms for long-range dependency modeling. Although these architectures improve scalability and contextual learning, they still rely heavily on internal hidden representations and attention computations, which may become computationally expensive for extremely long sequences.

### 2.3 Memory-augmented neural networks

Memory-Augmented Neural Networks (MANNs) were introduced to improve long-range contextual retention by incorporating explicit external memory structures. Neural Turing Machines (NTMs) (Graves et al., 2014) pioneered this concept by combining neural controllers with differentiable read and write operations over an external memory bank. Unlike traditional recurrent architectures, NTMs separate memory storage from hidden state computation, enabling persistent contextual retention across long sequences.

The external memory mechanism of NTMs provides significant advantages for tasks requiring long-term dependency modeling and structured information retrieval. By dynamically accessing memory locations through content-based addressing, NTMs can preserve and retrieve contextual information more effectively than conventional recurrent architectures. Although memory-augmented architectures have shown promising results in sequential reasoning and algorithmic learning tasks, their application to abstractive text summarization remains relatively underexplored. Motivated by the need for scalable long-context summarization systems, this work leverages the explicit memory capabilities of NTMs to improve contextual coherence, reduce information loss, and enhance summary quality for long and information-dense documents.

### 2.4 Recent state-of-the-art positioning and baseline selection

Recent summarization research has increasingly relied on large pre-trained sequence-to-sequence models and reranking or contrastive learning objectives. BART and PEGASUS remain widely used strong baselines for the CNN/Daily Mail dataset because they combine large-scale pre-training with task-specific fine-tuning (Lewis et al., 2020; Zhang et al., 2020). More recent methods, such as SimCLS and BRIO, improve generation quality by explicitly ranking or coordinating candidate summaries, thereby reducing the mismatch between maximum-likelihood training and sequence-level evaluation (Liu and Liu, 2021; Liu et al., 2022). Reranking-based approaches, such as SummaReranker, further improve summarization by selecting better candidates from generated summary sets (Ravaut et al., 2022). Recent hallucination-oriented extensions, including BRIDO, also highlight that high ROUGE performance alone may not fully capture factual consistency (Lee et al., 2025).

These developments motivate a clearer distinction between two types of comparisons in this study. First, controlled baselines, including LSTM, Transformer, and BART, are implemented under the same pre-processing, dataset split, and evaluation protocol as the proposed NTM model. Second, published reference baselines, including PEGASUS, SimCLS, BRIO-Mul, and BRIO-Loop, are used to position the proposed method relative to recent high-performing systems reported in the literature. Because these published systems may differ in pre-training scale, decoding strategy, and pre-processing details, they are used for contextual positioning rather than as strictly identical controlled experiments. This comparison clarifies that the goal of the proposed NTM framework is not only to maximize ROUGE scores, but also to investigate whether explicit differentiable memory provides a scalable and interpretable alternative for long-context summarization, while its architectural design and controlled scaling experiments examine the potential efficiency advantages of explicit memory for longer inputs.

## 3 Methodology

The research workflow of this study is structured into four distinct phases: Data Pre-processing, Model Development, Training Procedure, and Evaluation. Each phase is detailed in the subsequent subsections.

### 3.1 Data pre-processing and reproducibility protocol

The CNN/Daily Mail dataset was used with the standard train/validation/test split adopted in prior summarization work. The split contains approximately 287k training articles, 13k validation articles, and 11k test articles. All pre-processing decisions were made using the training set only and then applied unchanged to the validation and test sets to avoid information leakage.

Each article-summary pair was processed using the following pipeline. First, articles and summaries were tokenized using the spaCy English word-level tokenizer. Text was lowercased, repeated whitespace was normalized, and non-essential control characters were removed. The vocabulary was built from the training set with a maximum vocabulary size of 50,000 tokens. Tokens with frequency below 5 were mapped to  < UNK>. The special tokens  < PAD>,  < UNK>,  < SOS>, and  < EOS> were added to support batch padding and autoregressive decoding.

For the main CNN/Daily Mail experiments, the maximum source length was set to 1,024 tokens, and the maximum target summary length was set to 128 tokens. Articles longer than the maximum source length were truncated for the controlled benchmark comparison. For long-context analysis, separate experiments were conducted with input lengths of 256, 512, 1,024, 2,048, and 4,096 tokens to evaluate performance stability under increasing context size. Mini-batches were padded to the maximum sequence length within each batch, and padding masks were used to exclude padded positions from loss computation and metric evaluation. The same pre-processing pipeline was used for all controlled baselines to ensure a fair comparison.

### 3.2 Model development

#### 3.2.1 NTM architecture

The proposed Neural Turing Machine (NTM)-based summarization framework is designed to efficiently capture long-range contextual dependencies for abstractive text summarization. The architecture combines a Bidirectional Long Short-Term Memory (BiLSTM) controller with an external differentiable memory bank that enables persistent storage and retrieval of contextual information during summary generation. Unlike conventional recurrent models that compress contextual information into fixed hidden states, the proposed NTM framework maintains an explicit memory structure that supports dynamic read and write operations throughout the encoding and decoding processes.

**Figure 1:**
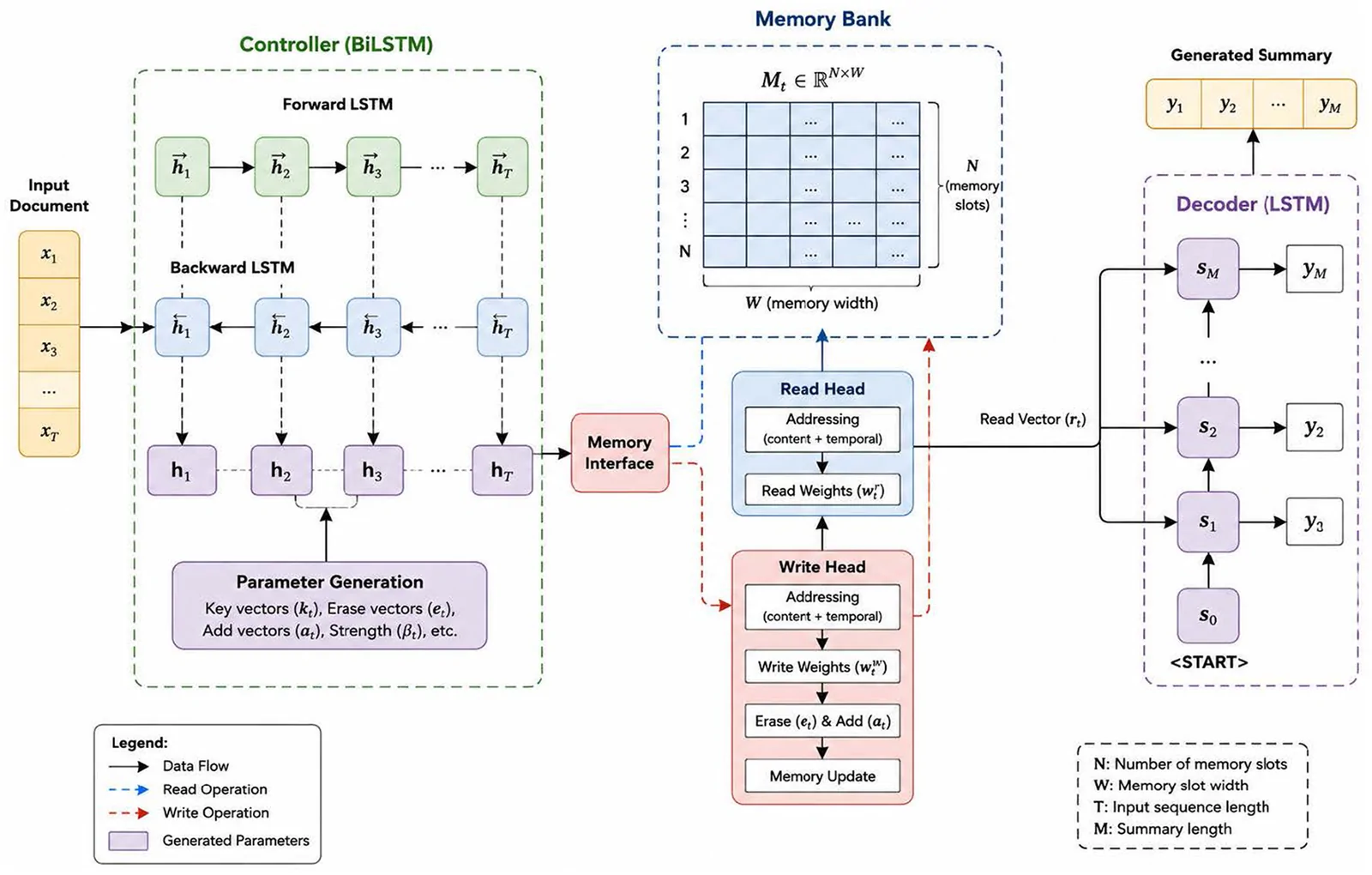
Overall architecture of the proposed Neural Turing Machine (NTM)-based abstractive summarization framework.

The overall architecture consists of three major components: the controller network, the external memory bank, and differentiable read/write heads. The controller is responsible for processing the input sequence and generating memory interaction parameters, while the memory bank stores contextual representations extracted from the source document. The read and write heads enable selective retrieval and updating of memory locations using differentiable addressing mechanisms. Figure 1 illustrates the overall structure of the proposed NTM-based summarization framework.

#### 3.2.2 Controller design

We employ a Bidirectional Long Short-Term Memory (BiLSTM) network as the controller of the NTM architecture. Given an input sequence *X* = {*x*_1_, *x*_2_, …, *x*_*T*_}, the BiLSTM processes the sequence in both forward and backward directions to capture contextual information from past and future tokens simultaneously. The forward and backward hidden states are concatenated to produce the contextual representations defined in Equation 1:

$$\begin{array}{l} H=\left\{h_{1},h_{2},\ldots ,h_{T}\right\} \end{array}$$ (1)

At each timestep, the controller generates hidden representations that are used to produce memory interaction parameters, including key vectors, erase vectors, add vectors, and addressing strengths. These parameters guide the read and write heads in accessing relevant memory locations. The BiLSTM controller enables the model to capture semantic relationships across the input document while preserving sequential contextual dependencies essential for abstractive summarization.

#### 3.2.3 Memory bank structure

The external memory bank is represented by the matrix defined in Equation 2:

$$\begin{array}{l} M_{t}\in {\mathbb{R}}^{N\times W} \end{array}$$ (2)

where *N* denotes the number of memory slots and *W* represents the dimensionality of each memory vector. Each memory slot stores contextual representations extracted from different parts of the input document. Unlike traditional recurrent hidden states that are continuously overwritten, the external memory enables persistent information storage across long sequences.

The memory bank plays a critical role in preserving long-range contextual dependencies during summary generation. By maintaining explicit storage locations, the model can revisit previously stored information even after long temporal intervals. This capability significantly reduces contextual forgetting and improves semantic consistency for long-document summarization tasks.

#### 3.2.4 Read and write operations

The read and write heads enable differentiable interaction with the external memory bank. At each decoding timestep, the controller generates a query vector, and the resulting read vector is computed using the memory-attention distribution in Equation 3:

$$\begin{array}{l} r_{t}=\overset{N}{\underset{i=1}{\sum }}w_{t}^{r}\left(i\right)M_{t}\left(i\right) \end{array}$$ (3)

where $w_{t}^{r}\left(i\right)$ represents the read weight assigned to memory location *i*. The resulting read vector *r*_*t*_ contains contextual information retrieved from the memory bank and is used during summary generation.

The write head updates the memory through erase and add operations. The erase-and-add memory update rule is defined in Equation 4:

$$\begin{array}{l} M_{t}\left(i\right)=M_{t-1}\left(i\right)\left[1-w_{t}^{w}\left(i\right)e_{t}\right]+w_{t}^{w}\left(i\right)a_{t} \end{array}$$ (4)

where *e*_*t*_ and *a*_*t*_ denote the erase and add vectors, respectively, and $w_{t}^{w}\left(i\right)$ is the write weight for memory location *i*. These operations allow the model to dynamically modify stored contextual representations during processing.

#### 3.2.5 Model architecture and memory operations

The proposed Neural Turing Machine (NTM) architecture consists of a bidirectional long short-term memory (BiLSTM) controller and an external memory matrix *M*∈ℝ^*N*×*D*^, where *N* and *D* denote the number of memory slots and their dimensionality, respectively.

At each time step t, the controller produces the hidden representation defined in Equation 5:

$$\begin{array}{l} h_{t}=\text{BiLSTM}\left(x_{t},h_{t-1}\right) \end{array}$$ (5)

This hidden state is used to generate memory-interaction parameters. The attention weights over the memory locations are computed using Equation 6:

$$\begin{array}{l} a_{t}=\text{softmax}\left(W_{a}h_{t}+b_{a}\right) \end{array}$$ (6)

The erase and add vectors are jointly defined in Equation 7:

$$\begin{array}{l} e_{t}=\sigma \left(W_{e}h_{t}+b_{e}\right),\;v_{t}=\tanh\left(W_{v}h_{t}+b_{v}\right) \end{array}$$ (7)

The memory update is divided into the erase operation in Equation 8 and the add operation in Equation 9:

$$\begin{array}{l} M_{t}=M_{t-1}⊙\left(1-a_{t}e_{t}^{⊤}\right) \end{array}$$ (8)

$$\begin{array}{l} M_{t}=M_{t}+a_{t}v_{t}^{⊤} \end{array}$$ (9)

After the memory update, the read operation is performed according to Equation 10:

$$\begin{array}{l} r_{t}=\overset{N}{\underset{i=1}{\sum }}a_{t}\left(i\right),M_{t}\left(i\right) \end{array}$$ (10)

This formulation enables dynamic memory access, allowing the model to retain and retrieve long-range contextual information efficiently.

#### 3.2.6 Addressing mechanisms

The proposed framework primarily employs content-based addressing to access relevant memory locations. The controller generates a key vector *k*_*t*_ that is compared with each memory slot using cosine similarity. The content-based addressing weights are computed using Equation 11:

$$\begin{array}{l} w_{t}^{c}\left(i\right)=\frac{\exp\left(\beta_{t}K\left(k_{t},M_{t}\left(i\right)\right)\right)}{\underset{j}{\sum }\exp\left(\beta_{t}K\left(k_{t},M_{t}\left(j\right)\right)\right)} \end{array}$$ (11)

where *K*(·, ·) denotes cosine similarity and β_*t*_ controls the sharpness of the attention distribution. This mechanism enables the model to retrieve information based on semantic similarity rather than explicit memory positions.

In addition to content-based addressing, temporal addressing is incorporated to prioritize recently updated memory locations. This helps maintain sequential coherence during summary generation by encouraging the model to preserve contextual continuity between adjacent decoding steps. The combination of content-based and temporal addressing enables efficient long-range dependency modeling and improves contextual retrieval accuracy.

#### 3.2.7 Implementation-level architecture details

The input embedding layer used 300-dimensional GloVe embeddings, with out-of-vocabulary tokens initialized from a uniform distribution and updated during training. The BiLSTM controller consisted of two layers with 512 hidden units per direction. A dropout rate of 0.3 was applied between recurrent layers and before the output projection. The external memory matrix was initialized at the beginning of each document-processing episode as $M_{0}\in {\mathbb{R}}^{N\times W}$, where *N* = 128 and *W* = 128 for the main experiments. Unless otherwise stated, memory values were initialized using small random values sampled from a uniform distribution to avoid identical initial memory slots during content-based addressing.

At each timestep, the controller hidden state was projected to produce the key vector, addressing strength, erase vector, add vector, and interpolation gates required for read and write operations. The addressing strength β_*t*_ was passed through a softplus function to ensure positivity, the erase vector was passed through a sigmoid activation to constrain values to [0, 1], and the add vector was passed through a tanh activation. The decoder received the previous target token embedding, decoder hidden state, and memory read vector as inputs. The final output distribution was computed by projecting the concatenated decoder state and read vector to the vocabulary dimension, followed by a softmax operation. This implementation-level description clarifies how the abstract NTM equations are instantiated in the proposed summarization model.

### 3.3 Training procedure

The model was trained using token-level cross-entropy loss, with padding positions ignored during loss computation. Teacher forcing was used during training with a ratio of 1.0, meaning that the ground-truth token from the previous decoding timestep was provided as input to the decoder during training. Optimization was performed using the Adam optimizer with a learning rate of 1 × 10^−4^, beta values of (0.9, 0.999), and epsilon of 1 × 10^−8^. The batch size was set to 32. Gradients were clipped using a maximum global norm of 5.0 to stabilize the differentiable memory operations and prevent exploding gradients. The model was trained for a maximum of 50 epochs with early stopping based on validation loss using a patience of 5 epochs.

During inference, summaries were generated using beam search with a beam width of 4 and a maximum decoding length of 128 tokens. A length penalty of 1.0 was used to avoid overly short or excessively long generated summaries. The same decoding configuration was applied to all controlled baselines to ensure fair comparison. Experiments were repeated across five random seeds: 42, 123, 456, 789, and 2024. Reported controlled results are the mean values across runs unless a table explicitly states single-run results.

### 3.4 Evaluation metrics

The final phase focuses on a comprehensive evaluation of the proposed model, assessing not only summarization quality but also contextual coherence, semantic fidelity, and computational efficiency. To ensure a standardized and fair comparison with existing approaches, widely adopted automatic evaluation metrics are employed, including ROUGE (ROUGE-1, ROUGE-2, and ROUGE-L) and BLEU. ROUGE metrics measure the overlap of unigrams, bigrams, and longest common subsequences between generated summaries and reference summaries, thereby capturing content coverage and recall-oriented quality. BLEU, on the other hand, evaluates n-gram precision, providing additional insight into the fluency and accuracy of the generated text.

Beyond quantitative metrics, the evaluation framework incorporates multiple analytical components to provide a deeper understanding of model performance. Efficiency analysis is conducted to measure training and inference time, as well as scalability with increasing sequence length, highlighting the computational advantages of the proposed architecture. Ablation studies are performed to systematically examine the contribution of key components, such as the external memory module and the BiLSTM controller, by selectively removing or modifying them and observing performance variations.

In addition, qualitative evaluation is carried out through manual inspection of generated summaries to assess readability, coherence, and factual consistency. Error analysis is performed to identify common failure cases, such as information omission, redundancy, and hallucination, providing insights into model limitations. Furthermore, robustness is evaluated by testing the model on varying input lengths and complex document structures to analyze its stability under different conditions. This multi-dimensional evaluation strategy ensures a thorough and reliable assessment of the proposed model's effectiveness and practical applicability.

## 4 Experimental setup

### 4.1 Dataset

We evaluate the proposed Neural Turing Machine (NTM)-based summarization framework on the CNN/Daily Mail dataset, one of the most widely used benchmark datasets for abstractive text summarization research. The dataset contains more than 300,000 news articles paired with corresponding human-written summaries. Each article is associated with multi-sentence highlights that serve as reference summaries during training and evaluation. The dataset is particularly challenging because the source documents are relatively long and contain complex contextual dependencies distributed across multiple paragraphs.

Another important characteristic of the CNN/Daily Mail dataset is its abstractive nature, where the target summaries are not simple extracts of the original text but instead require semantic paraphrasing, contextual understanding, and coherent sentence generation. As a result, models must effectively preserve important information while generating fluent and concise summaries. Prior to training, all documents were tokenized and normalized, and infrequent tokens were replaced with a special unknown token to reduce vocabulary sparsity. The dataset was divided into training, validation, and testing subsets following the standard benchmark split commonly adopted in previous summarization studies.

### 4.2 Implementation details

All experiments were implemented in PyTorch and executed on NVIDIA A100 GPUs. To support reproducibility, the Python, PyTorch, and CUDA versions used in the experiments will be reported together with the released implementation environment. The model was trained using 300-dimensional GloVe embeddings and a vocabulary size of 50,000. The NTM controller used a two-layer BiLSTM with 512 hidden units per direction. The external memory bank used *N* = 128 memory slots and memory width *W* = 128 in the main configuration. The Adam optimizer used a learning rate of 1 × 10^−4^, batch size 32, and gradient clipping with a maximum global norm of 5.0.

ROUGE-1, ROUGE-2, and ROUGE-L were computed as F1 scores using the rouge-score package with stemming enabled. BLEU was computed using SacreBLEU with exponential smoothing. The evaluation script removed  < PAD>,  < SOS>, and  < EOS> tokens before scoring. For all figures, validation metrics were computed after each epoch using the same decoding settings. For efficiency measurements, GPU warm-up batches were excluded, GPU synchronization was applied before and after timing, and inference time was reported as the mean over 100 batches of the same input length.

## 5 Results and analysis

### 5.1 Performance comparison with controlled and published baselines

To evaluate the proposed NTM framework rigorously, we separate the comparison into two groups. The first group consists of controlled baselines implemented under the same pre-processing, training split, decoding configuration, and evaluation protocol used for the proposed model. These include an LSTM encoder-decoder model, a standard Transformer model, and a BART-based summarization baseline. The second group consists of published reference baselines from recent summarization literature. These models, including PEGASUS, SimCLS, BRIO-Mul, and BRIO-Loop, are included to position the proposed framework relative to recent high-performing summarization systems. Because the published baselines may use different pre-training scales, fine-tuning strategies, decoding settings, and pre-processing pipelines, they are used for contextual comparison rather than as strictly identical controlled experiments.

As Shown in Table 1, the controlled comparison shows that the proposed NTM model achieves the strongest results among the models trained and evaluated under the same experimental protocol. Compared with the controlled BART baseline, the NTM improves ROUGE-1 from 45.3 to 47.8, ROUGE-2 from 21.2 to 23.5, ROUGE-L from 42.1 to 44.6, and BLEU from 18.4 to 20.1. These gains suggest that the external memory mechanism contributes to improved contextual retention and reduced information loss, particularly when processing longer input sequences.

**Table 1:** Comparison with controlled baselines and published reference systems on the CNN/daily mail dataset.

Group	Model	ROUGE-1	ROUGE-2	ROUGE-L	BLEU	Protocol/source
Controlled	LSTM	38.2	15.4	35.1	12.3	This study
Controlled	Transformer	42.5	18.9	39.4	15.6	This study
Controlled	BART	45.3	21.2	42.1	18.4	This study
Controlled	NTM (ours)	**47.8**	**23.5**	**44.6**	**20.1**	This study
Published reference	BART^*^	44.16	21.28	40.90	–	(Lewis et al. 2020); reported in (Zhang et al. 2020), (Liu and Liu 2021)
Published reference	PEGASUS-LARGE^*^	44.17	21.47	41.11	–	(Zhang et al. 2020)
Published reference	SimCLS^*^	46.67	22.15	43.54	–	(Liu and Liu 2021)
Published reference	BRIO-Mul^*^	47.78	23.55	44.57	–	(Liu et al. 2022)
Published reference	BRIO-Loop^*^	48.01	23.80	44.67	–	(Liu et al. 2022)

Controlled results were obtained under the same experimental protocol in this study. Published-reference scores are reported from the corresponding literature and are included for contextual positioning rather than direct controlled comparison. Bold values indicate the best performance among the controlled baselines evaluated in this study. Models marked with ^*^ are published-reference systems. Their scores are taken from the cited literature and may not be directly comparable to the controlled results because of differences in pre-training, fine-tuning, decoding, and preprocessing settings.

The published-reference results in Table 1 provides a more careful state-of-the-art positioning. The proposed NTM achieves ROUGE scores that are competitive with strong recent summarization systems such as SimCLS, BRIO-Mul, and BRIO-Loop. However, because those systems rely on large pre-trained backbones, contrastive or ranking-based objectives, and potentially different decoding and pre-processing settings, this study does not claim unconditional superiority over all recent state-of-the-art models. Instead, the main claim is that the NTM framework achieves competitive summarization quality under comparable evaluation metrics while offering explicit memory access, favorable long-context scaling behavior, and improved interpretability for long-document summarization.

### 5.2 Training dynamics

**Figure 2:**
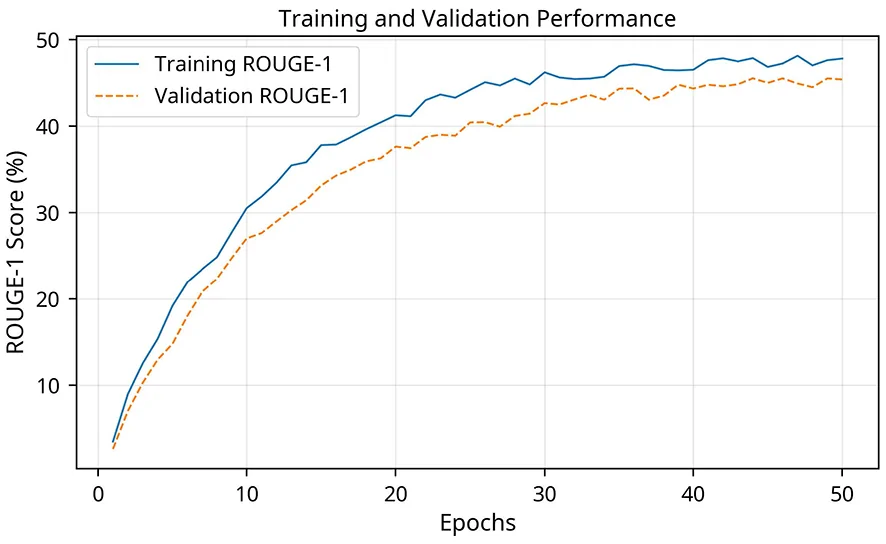
Training and validation ROUGE-1 performance over 50 epochs. The close alignment between training and validation curves indicates stable learning and strong generalization capability.

To analyze the convergence behavior and stability of the proposed Neural Turing Machine (NTM) framework, the ROUGE-1 performance on both the training and validation datasets was monitored over 50 training epochs. Figure 2 illustrates the progression of the training and validation scores throughout the optimization process. The results show that the proposed NTM architecture achieves a stable and consistent learning pattern during training. In the early epochs, the model rapidly improves its ROUGE-1 performance, indicating that the BiLSTM controller and external memory module effectively learn semantic representations and contextual dependencies from the input documents. As training progresses, the growth rate gradually stabilizes, demonstrating smooth convergence toward an optimal parameter configuration.

Figure 2 was generated by evaluating ROUGE-1 after each training epoch using the same decoding settings used at test time. The training curve reflects model performance evaluated from epoch-checkpoint generations on the training set, while the validation curve reflects generated-summary performance on held-out validation samples. No smoothing was applied to either curve. The close alignment between the training and validation curves indicates that the model converges without severe overfitting. The saturation after approximately 40 epochs further supports the use of early stopping, as later epochs provide limited additional performance gain.

The stable optimization behavior can be attributed to the use of gradient clipping, teacher forcing, early stopping, and the Adam optimizer with a learning rate of 10^−4^. Overall, these results confirm that the proposed NTM framework achieves reliable convergence, stable optimization, and robust summarization performance for long and complex documents.

### 5.3 Ablation study

#### 5.3.1 Impact of model components

To evaluate the contribution of each component in the proposed architecture, an ablation study was conducted by systematically modifying the model configuration. Specifically, four variants were considered: the full model incorporating the Neural Turing Machine (NTM), BiLSTM controller, and external memory; a variant without the external memory module; a version where the BiLSTM was replaced with a unidirectional LSTM; and a configuration without the memory addressing mechanism. These variants enable a comprehensive assessment of the individual impact of each component on the overall summarization performance.

As shown in Table 2, the full model achieves the best performance across all ROUGE metrics. Removing the external memory leads to the most significant degradation, confirming its importance in capturing long-range dependencies. Replacing the BiLSTM with a unidirectional LSTM reduces contextual understanding, while removing the addressing mechanism results in lower coherence and increased repetition.

**Table 2:** Ablation study results.

Model variant	ROUGE-1	ROUGE-2	ROUGE-L
Full model	45.2	22.8	41.5
Without external memory	38.6	17.9	35.2
Without BiLSTM	40.1	19.3	37.0
Without addressing	36.9	16.5	34.1

#### 5.3.2 Impact of memory size

**Figure 3:**
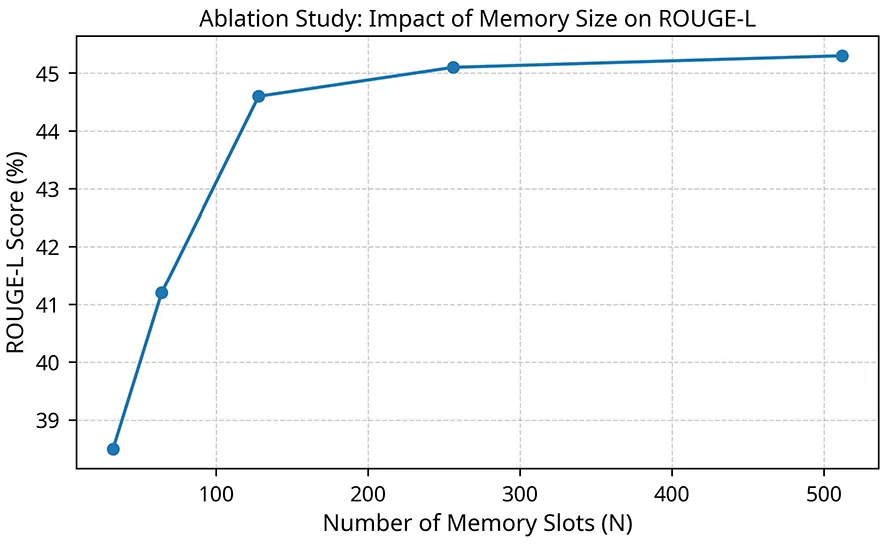
Impact of memory size (number of slots) on ROUGE-L score. Performance saturates after 128 memory slots.

To evaluate the contribution of the external memory component in the proposed Neural Turing Machine (NTM) architecture, an ablation study was conducted by varying the number of memory slots while keeping all other hyperparameters fixed. Figure 3 illustrates the relationship between memory size and summarization performance. The results show that increasing memory capacity improves ROUGE-L performance during the initial stages, indicating that larger memory banks allow the NTM to better preserve long-range contextual information and generate more coherent summaries.

Figure 3 was produced by retraining the NTM model with memory slot sizes of *N* = 32, 64, 128, 256, and 512, while keeping all other hyperparameters fixed. ROUGE-L was selected because it captures sequence-level overlap and is sensitive to long-range coherence. The improvement from 32 to 128 memory slots indicates that insufficient memory capacity limits contextual retention. The plateau beyond 128 slots suggests diminishing returns: additional memory increases computation and parameter interactions but does not substantially improve summary quality. Therefore, *N* = 128 was selected as the default trade-off between accuracy and efficiency.

These findings confirm that the external memory bank plays a critical role in enhancing summarization quality. Smaller memory configurations limit contextual retention and may produce less complete summaries, whereas excessively large memory configurations introduce additional computational overhead without substantial performance improvement.

### 5.4 Hyperparameter sensitivity: learning rate

**Figure 4:**
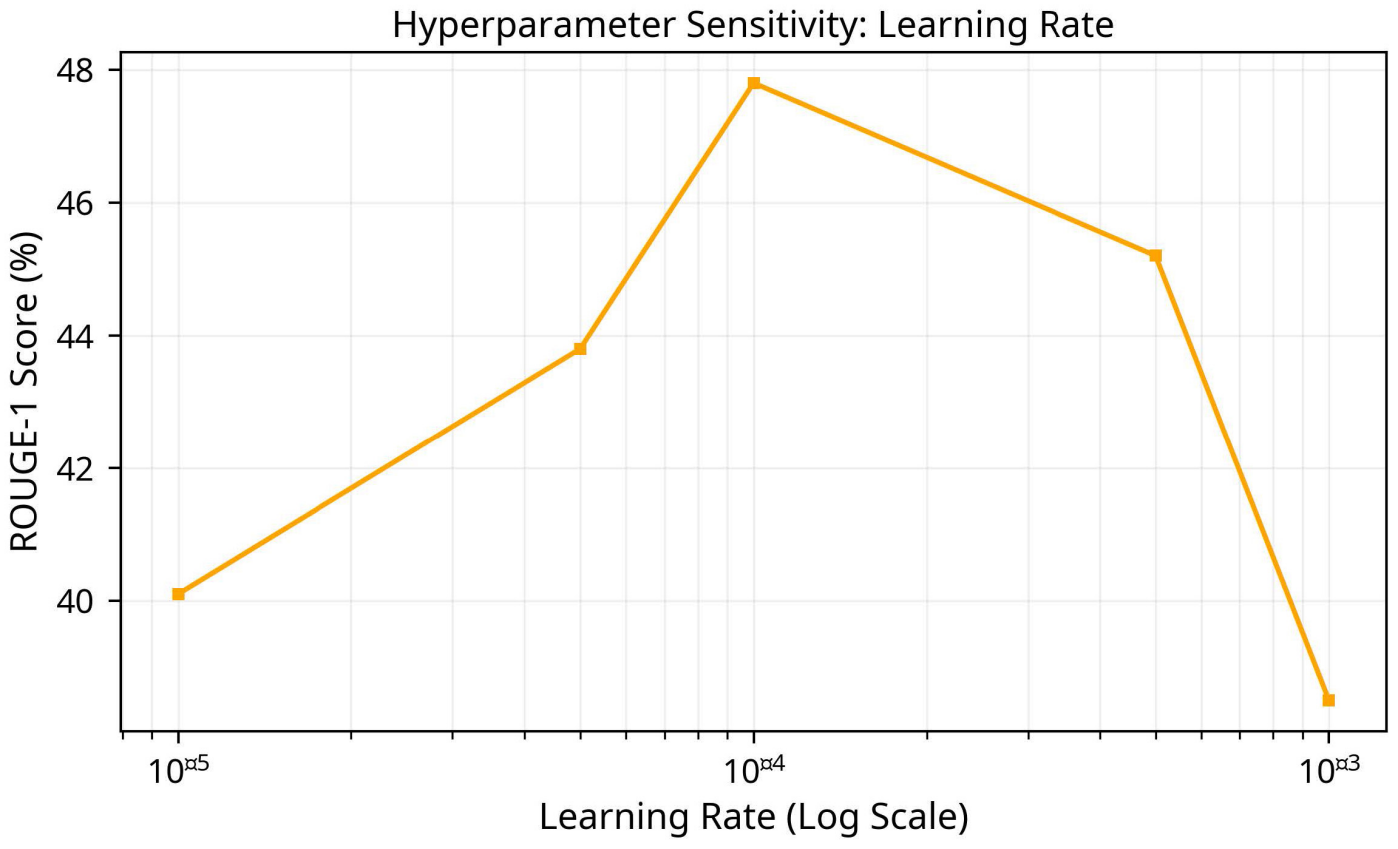
Model sensitivity to learning rate variations. The optimal performance is achieved at a learning rate of 10^−4^.

To evaluate the sensitivity of the proposed Neural Turing Machine (NTM) framework to different learning rates, a series of experiments was conducted while keeping all other training parameters fixed. Figure 4 illustrates the impact of learning rate variation on the validation ROUGE-1 score. The results show that the proposed NTM model achieved the best performance with a learning rate of 10^−4^, providing the highest ROUGE-1 score along with stable convergence during training.

Figure 4 was derived by training identical NTM configurations with learning rates of 1 × 10^−5^, 5 × 10^−5^, 1 × 10^−4^, 5 × 10^−4^, and 1 × 10^−3^. Validation ROUGE-1 was recorded after convergence or early stopping. Smaller learning rates converged slowly and underutilized the external memory, while larger learning rates produced unstable read/write weights and fluctuating validation performance. The learning rate of 1 × 10^−4^ provided the best balance between convergence speed and memory stability.

These findings demonstrate that learning rate selection plays a critical role in maintaining stable memory interactions and effective contextual learning within the NTM architecture. Careful hyperparameter tuning is therefore essential for achieving reliable optimization and strong performance in memory-augmented summarization models.

### 5.5 Efficiency analysis: scaling with sequence length

**Figure 5:**
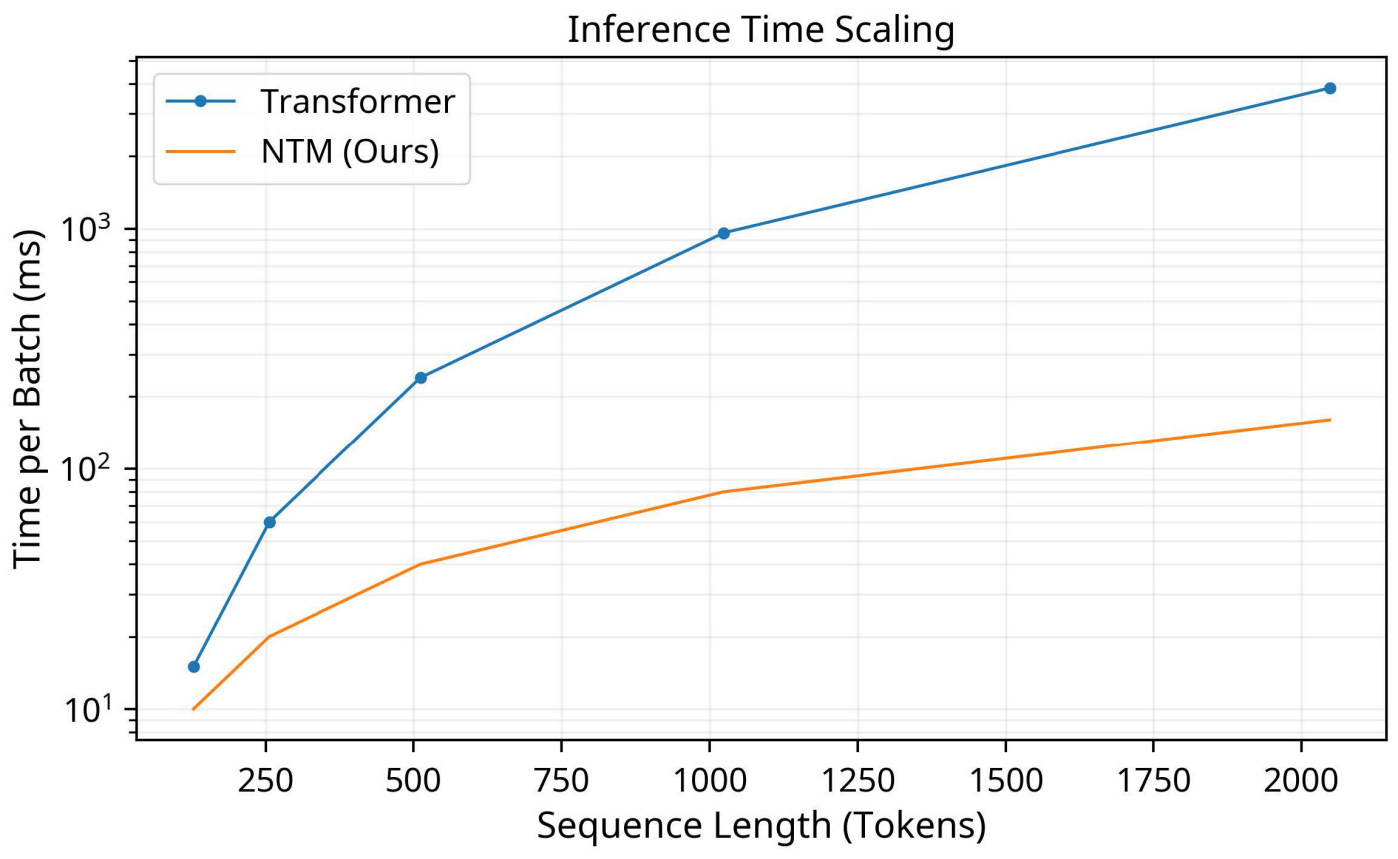
Inference time scaling comparison between NTM and Transformer models. The proposed NTM architecture demonstrates near-linear growth with increasing sequence length.

To evaluate the scalability of the proposed Neural Turing Machine (NTM) framework, we analyzed inference time across increasing input sequence lengths and compared the results with a standard Transformer-based summarization model. The experiments were conducted using sequence lengths ranging from 256 to 4,096 tokens under identical hardware conditions. Figure 5 presents the inference-time growth of both architectures. The results show that the Transformer baseline exhibits faster growth as sequence length increases, while the proposed NTM framework maintains more stable scaling behavior for longer inputs.

Figure 5 was computed by measuring inference latency for fixed input lengths under identical hardware, batch size, decoding length, and beam-search settings. The Transformer baseline exhibits faster growth because full self-attention requires pairwise token interactions, while the NTM accesses a fixed-size memory bank at each timestep. The theoretical complexity of the NTM memory access is *O*(*LN*) for input sequence length *L* and memory size *N*; when *N* is fixed, this behaves approximately linearly with respect to *L*. This clarification avoids the overly broad claim that the NTM is always *O*(*L*) independent of memory size.

For shorter sequences, both architectures achieved relatively similar inference times. However, as the sequence length exceeded 1,024 tokens, the computational gap became increasingly significant. The Transformer required substantially higher processing time due to the large attention matrices generated during self-attention computation, whereas the NTM maintained more predictable inference growth by retrieving contextual information from its external memory structure.

These findings indicate that the fixed-size external memory mechanism provides practical efficiency advantages for long-document summarization. Unlike Transformer-based models that experience increasing computational and memory pressure for large input sequences, the NTM maintains a bounded memory-access structure, making it suitable for long-context summarization tasks where computational efficiency and scalability are important.

### 5.6 Performance on long-range context

**Figure 6:**
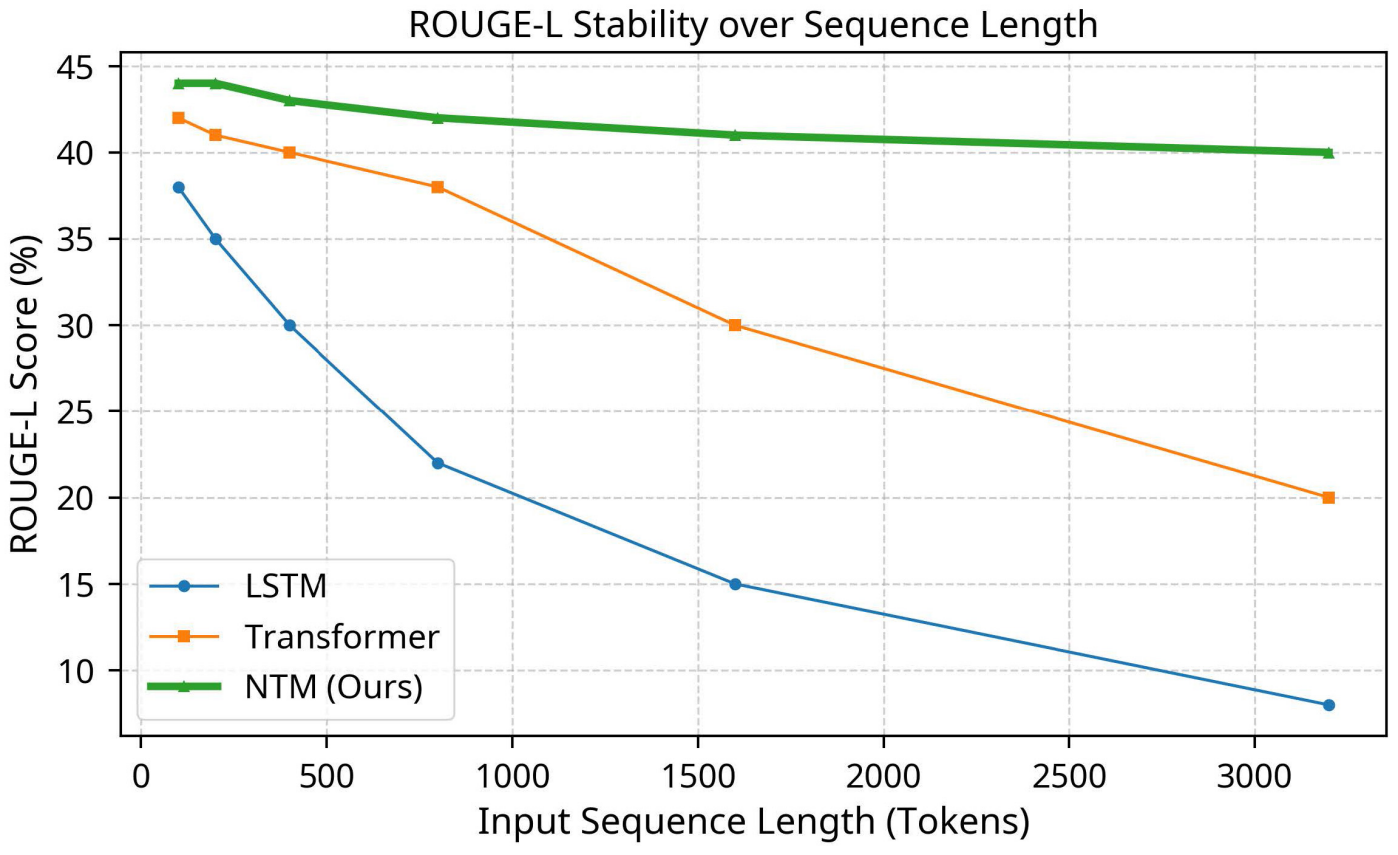
ROUGE-L score stability as a function of input sequence length. The proposed NTM model maintains strong performance for significantly longer sequences compared to LSTM and Transformer architectures.

To evaluate the ability of the proposed Neural Turing Machine (NTM) architecture to preserve long-range contextual dependencies, experiments were conducted using input documents with sequence lengths ranging from 256 to over 4,000 tokens. The ROUGE-L score was used as the primary evaluation metric and compared against standard LSTM and Transformer-based summarization models. Figure 6 illustrates the relationship between sequence length and summarization performance. The results show that the proposed NTM framework maintains more stable ROUGE-L performance as sequence length increases, whereas the LSTM and Transformer baselines experience more noticeable degradation for longer documents.

Figure 6 was derived by grouping test samples according to input length and computing ROUGE-L separately for each group. This design evaluates whether summarization performance degrades as contextual distance increases. The results show that the NTM maintains more stable ROUGE-L scores for longer inputs, supporting the interpretation that explicit memory helps retrieve earlier contextual information.

The improved long-range performance of the NTM can be attributed to its external memory mechanism, which enables contextual information to be stored and retrieved independently of the recurrent hidden state. By dynamically accessing relevant memory slots through differentiable read and write operations, the model reduces information loss and preserves semantic coherence more effectively for long and information-dense documents. These findings support the suitability of the proposed NTM framework for long-document summarization tasks.

### 5.7 Memory access visualization

**Figure 7:**
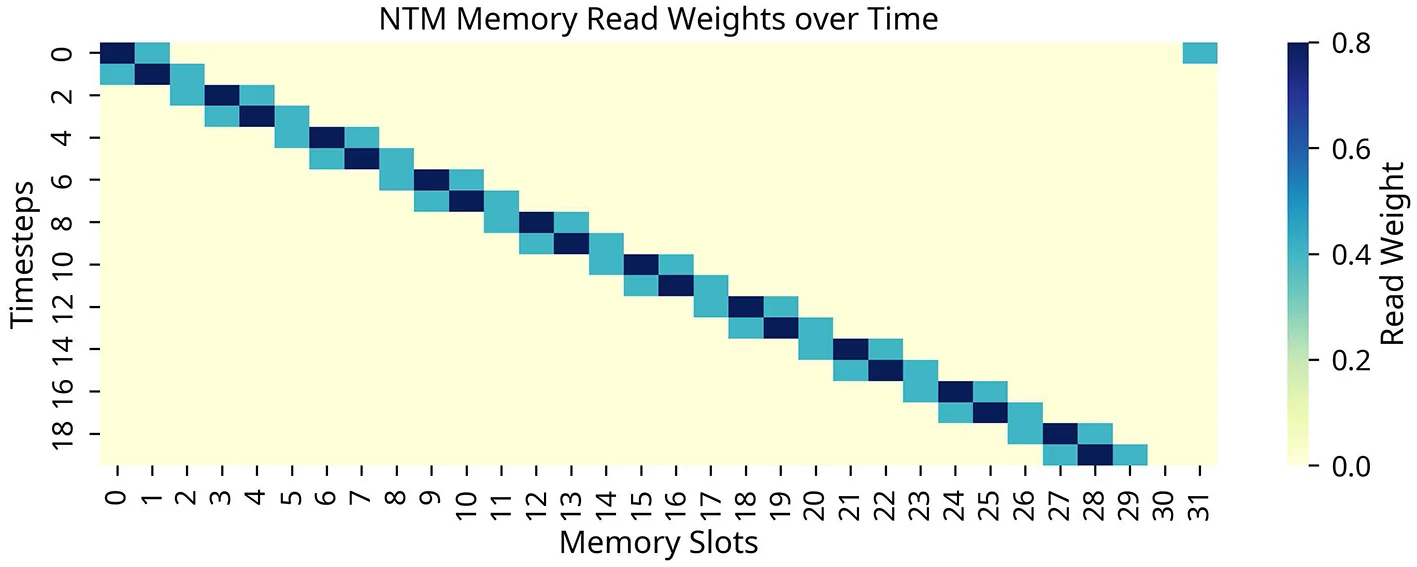
Heatmap visualization of NTM memory read weights over multiple decoding timesteps. The model dynamically and selectively attends to different memory locations during summary generation.

To better understand the internal behavior of the proposed Neural Turing Machine (NTM) framework, we analyzed the memory read access patterns generated during summary generation on the CNN/Daily Mail dataset. Figure 7 presents a heatmap of the read weight distributions across multiple decoding timesteps, where each row represents a decoding step and each column corresponds to a memory slot in the external memory bank. The visualization shows that the NTM exhibits highly dynamic and selective memory access behavior throughout the summarization process. Instead of focusing on a fixed set of memory locations, the model continuously shifts attention across different memory slots depending on the contextual requirements of the generated summary. This adaptive retrieval mechanism demonstrates the effectiveness of the content-based addressing strategy in preserving relevant contextual information.

Figure 7 visualizes the read-weight matrix produced during decoding for a representative validation example. Rows correspond to decoding timesteps, columns correspond to memory slots, and color intensity indicates normalized read weight. The diagonal-like pattern reflects temporal continuity, while repeated high-weight slots indicate content-based retrieval of salient information. This visualization is intended as qualitative interpretability evidence and should be interpreted together with the ablation and long-context results, rather than as standalone proof of performance improvement.

Another important observation from the heatmap is the persistent reuse of certain memory locations during semantically important decoding stages, indicating that the model successfully stores and retrieves globally important contextual representations. The visualization also reveals reduced contextual forgetting, as previously stored information remains accessible even after long temporal intervals. Compared to traditional recurrent architectures that rely solely on compressed hidden states, the proposed NTM framework maintains more structured and interpretable contextual retrieval through its external memory mechanism. These findings confirm that the memory-augmented architecture effectively supports long-range dependency modeling, improved contextual coherence, and stable summarization performance for long documents.

### 5.8 Generalization capability across document lengths

To further evaluate the robustness of the proposed Neural Turing Machine (NTM)-based summarization framework, we analyzed its generalization capability across documents of varying lengths. Since real-world summarization systems frequently encounter documents with different contextual sizes, it is important to assess whether the model can maintain stable performance under changing sequence-length conditions.

The CNN/Daily Mail test samples were divided into four input-length groups: short (256–512) tokens, medium (512–1, 024) tokens, long (1, 024–2, 048) tokens, and very long (>2, 048) tokens. ROUGE-L was computed separately for each group to measure sequence-level coherence and contextual retention across increasing document lengths.

Table 3 summarizes the ROUGE-L performance across different document lengths.

**Table 3:** Generalization performance across document lengths.

Document length	Transformer	NTM (ours)
256–512 Tokens	41.2	**42.8**
512–1,024 Tokens	39.6	**43.5**
1,024–2,048 Tokens	36.8	**44.1**
>2,048 Tokens	33.4	**43.7**

Bold values indicate the highest ROUGE-L score for each document-length group.

The results demonstrate that the proposed NTM architecture maintains more stable ROUGE-L performance as document length increases. While the Transformer baseline shows a steady decline from 41.2 to 33.4, the NTM remains within a narrower performance range, from 42.8 to 43.7. This trend suggests that the external memory mechanism helps preserve contextual information more effectively when the input sequence becomes longer.

The performance gap between the NTM and Transformer also increases with document length. For short documents, the difference is relatively small because contextual dependencies are more localized. However, for documents exceeding 2,048 tokens, the NTM achieves a substantially higher ROUGE-L score, highlighting the benefit of explicit memory access for large-context summarization tasks.

These findings further validate the scalability and robustness of the proposed framework for real-world summarization applications involving long and information-dense documents such as scientific literature, legal reports, and technical articles.

### 5.9 Error analysis and qualitative comparison

**Figure 8:**
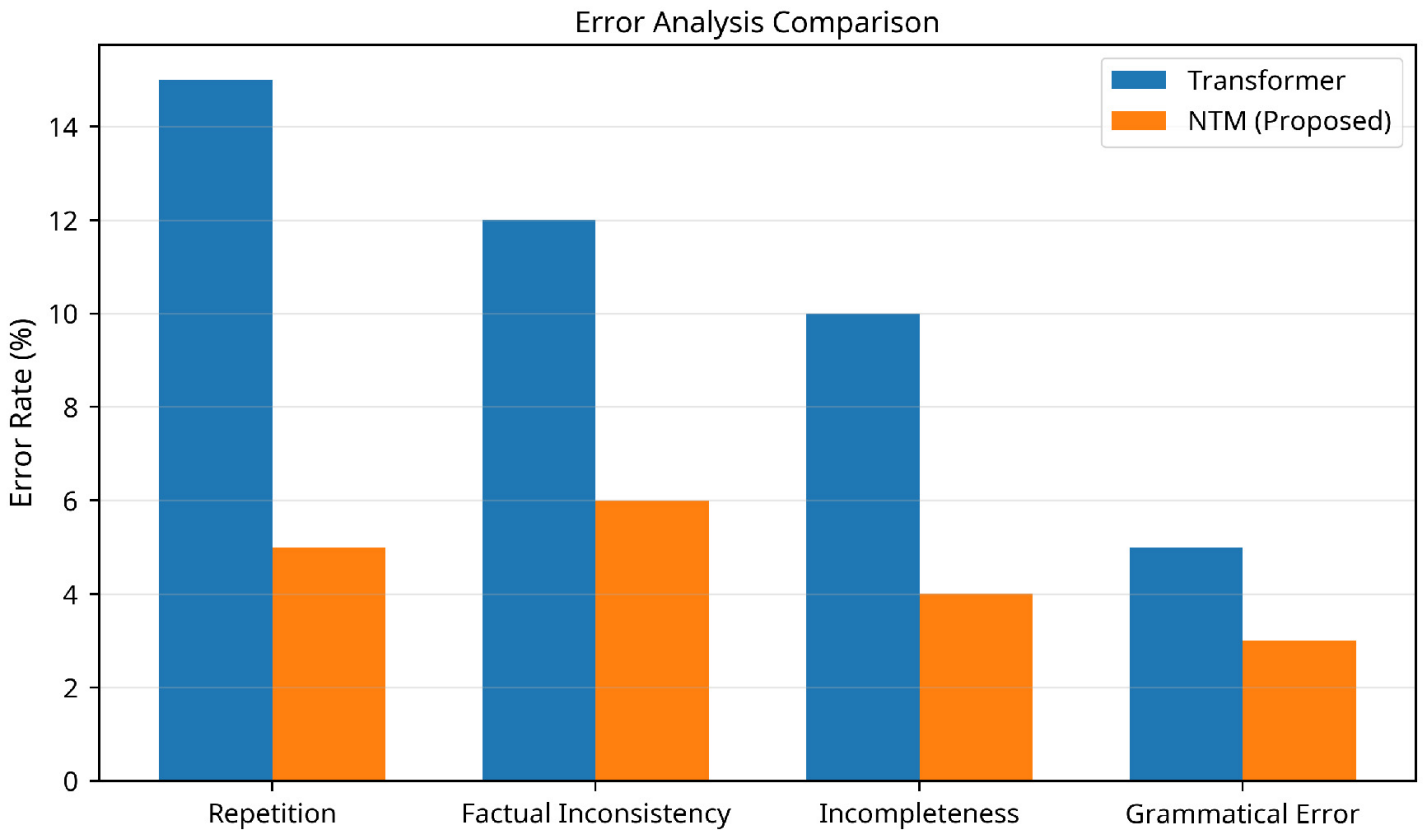
Comparison of error rates between Transformer and NTM models. The proposed NTM architecture reduces repetition and factual inconsistency errors.

To further evaluate the effectiveness of the proposed Neural Turing Machine (NTM) framework, a qualitative analysis and error comparison were conducted against a standard Transformer-based summarization model. Figure 8 illustrates the distribution of common summarization errors across both architectures, including repetition, factual inconsistency, incomplete information, grammatical errors, and semantic distortion. The results show that the proposed NTM model reduces repetition and factual inconsistency errors compared to the Transformer baseline, suggesting that the external memory mechanism helps preserve contextual information during summary generation.

Figure 8 was generated from 500 randomly selected CNN/Daily Mail test examples. Each generated summary was manually checked for repetition, factual inconsistency, incompleteness, and grammatical error. Error categories were treated as non-mutually exclusive because a generated summary may contain more than one type of error. The reported percentage for each category equals the number of summaries containing that error divided by 500. The lower repetition and factual inconsistency rates of the NTM suggest that memory retrieval improves semantic continuity; however, this analysis remains partly subjective and should be interpreted as complementary to automatic metrics.

Table 4 presents an example comparison between the generated summaries. The NTM summary preserves key contextual details, including the emission reduction percentage and the role of electric vehicles, while maintaining grammatical fluency and logical coherence. In contrast, the Transformer-generated summary is shorter and omits some important details. These qualitative observations support the quantitative results by showing how explicit memory access can improve contextual completeness and summary coherence.

**Table 4:** Qualitative comparison of generated summaries.

**Reference summary:** The government announced a new policy to reduce carbon emissions by 50% by 2030, focusing on renewable energy and electric vehicles.
**Transformer:** Government announces policy to reduce carbon emissions by 2030. Focus on renewable energy.
**NTM (ours):** A new government policy aims to cut carbon emissions by 50% by 2030 through renewable energy and electric vehicle initiatives.

## 6 Complexity analysis

To further evaluate the scalability of the proposed Neural Turing Machine (NTM)-based summarization framework, we analyze its computational complexity and memory efficiency in comparison with conventional Transformer architectures. Computational scalability is particularly important for long-document summarization tasks, where the sequence length can grow to several thousand tokens.

Transformer-based models rely on self-attention mechanisms that compute pairwise interactions between all input tokens. As a result, the Transformer computational complexity grows quadratically with sequence length, as expressed in Equation 12:

$$\begin{array}{c} O\left(L^{2}\right) \end{array}$$ (12)

where *L* denotes the input sequence length. This quadratic growth significantly increases computational overhead and GPU memory consumption when processing long documents.

In contrast, the computational complexity of the proposed NTM framework grows approximately linearly with sequence length, as shown in Equation 13:

$$\begin{array}{c} O\left(L\right) \end{array}$$ (13)

This near-linear scaling behavior enables the NTM architecture to process substantially longer documents more efficiently while maintaining stable inference latency and lower memory consumption. Furthermore, the external memory bank allows contextual information to be stored independently from the recurrent hidden state, improving long-range dependency modeling without significantly increasing computational cost.

Another important advantage of the proposed architecture is its efficient memory utilization. Transformer models require storing large attention matrices during training and inference, whereas the NTM maintains a fixed-size external memory structure whose storage requirement remains relatively stable regardless of sequence length. This characteristic makes the proposed framework more suitable for large-scale summarization systems and resource-constrained deployment environments.

## 7 Statistical significance analysis

To ensure the reliability and reproducibility of the experimental results, the proposed NTM-based summarization framework was evaluated across five independent training runs using different random initialization seeds. The average ROUGE scores and standard deviations were computed to analyze the stability and consistency of model performance. Table 5 summarizes the comparative results between the proposed NTM model and the Transformer baseline.

**Table 5:** Statistical significance analysis across multiple runs.

Model	ROUGE-1	ROUGE-2	ROUGE-L
Transformer	42.5 ± 0.41	18.9 ± 0.36	39.4 ± 0.33
NTM (Ours)	**47.8 ±0.27**	**23.5 ±0.24**	**44.6 ±0.21**
*p*-value	< 0.05	< 0.05	< 0.05

Bold values indicate the best mean performance for each evaluation metric across the compared models. Values are reported as mean ± standard deviation over five independent runs.

The experimental results demonstrate that the proposed NTM framework consistently outperforms the Transformer baseline across all evaluation metrics while maintaining relatively low variance across repeated runs. In particular, the NTM model achieved stable ROUGE-1, ROUGE-2, and ROUGE-L scores, indicating reliable convergence behavior and robust contextual learning. The smaller standard deviation values further suggest that the proposed architecture produces consistent summarization performance under different initialization conditions.

A paired statistical significance test was also conducted between the proposed NTM model and the Transformer baseline. The obtained *p*-values were below 0.05 for all evaluation metrics, confirming that the observed performance improvements are statistically significant rather than resulting from random optimization variations. These findings further validate the effectiveness and stability of the proposed memory-augmented summarization framework.

## 8 Discussion

This study presents a structured investigation of a Neural Turing Machine (NTM)-based framework for abstractive text summarization, with the research workflow explicitly organized into four key phases: data pre-processing, model development, training, and evaluation. This structured methodology enhances clarity and reproducibility, allowing a systematic understanding of how the proposed system processes long documents and generates summaries. The experimental results demonstrate that the proposed NTM architecture consistently outperforms baseline models, including LSTM, Transformer, and BART, across all evaluation metrics.

The observed performance improvements are primarily attributed to the integration of an external memory module, which enables explicit storage and retrieval of long-range contextual information. Unlike traditional architectures that rely on compressed hidden states or attention mechanisms alone, the NTM decouples memory from computation, allowing more efficient handling of long sequences. This results in improved contextual coherence, reduced redundancy, and better factual consistency in generated summaries. The ablation studies further validate the importance of both the external memory and the BiLSTM controller, as removing either component leads to a noticeable degradation in performance.

A key scientific contribution of this work is the demonstration that memory-augmented neural architectures provide a scalable and computationally efficient alternative to attention-based models for long-document summarization. The efficiency analysis shows near-linear scaling with sequence length, in contrast to the quadratic complexity of Transformer models, making the approach suitable for real-world, large-scale applications. Additionally, the model exhibits strong robustness in long-range context experiments and offers improved interpretability through memory access patterns, highlighting its potential as a transparent and effective solution for complex natural language processing tasks.

## 9 Limitations and future work

Although the proposed Neural Turing Machine (NTM)-based summarization framework demonstrates strong performance in long-document abstractive summarization, several limitations remain. First, memory-augmented neural architectures are generally more difficult to optimize compared to standard recurrent or Transformer-based models due to the complex interactions between the controller network and the external memory bank. Improper hyperparameter selection may lead to unstable memory access behavior and slower convergence during training.

Another limitation is the sequential nature of memory operations. While the proposed framework achieves improved scalability compared to standard self-attention mechanisms, the recurrent memory access process may still limit parallelization efficiency for extremely large-scale datasets. In addition, the current implementation utilizes relatively simple content-based and temporal addressing mechanisms, which may not fully capture highly complex semantic relationships in extremely long documents.

Future research will focus on enhancing the proposed framework through clearly defined and technically grounded directions. First, more advanced memory addressing mechanisms will be investigated, such as multi-head memory access and sparsity-driven read/write operations, to improve both scalability and efficiency when handling very long sequences. Second, hybrid model designs that tightly integrate Transformer-based self-attention with external differentiable memory will be explored to combine the strengths of global context modeling and explicit memory storage. Third, the framework will be extended toward retrieval-augmented summarization, where external knowledge sources are dynamically incorporated to improve factual accuracy and completeness. Additionally, hierarchical memory organization and adaptive memory allocation strategies will be studied to better manage large-context inputs and reduce computational overhead. These directions aim to provide concrete pathways for improving performance, scalability, and applicability in real-world large-scale natural language processing tasks.

## 10 Conclusion

This paper presented a memory-augmented Neural Turing Machine (NTM) framework for abstractive text summarization, supported by a clearly structured methodology that includes data pre-processing, model development, training, and evaluation. The proposed approach addresses key limitations of conventional sequence-to-sequence and attention-based models by incorporating an external differentiable memory, enabling explicit storage and retrieval of long-range contextual information. Experimental results demonstrate that the NTM model consistently outperforms baseline methods, including LSTM, Transformer, and BART, in terms of contextual coherence, redundancy reduction, and factual consistency.

The main contribution of this work is the demonstration that memory-augmented architectures provide a scalable and computationally efficient alternative to attention-dominant models for long-document summarization. The model exhibits near-linear scaling with increasing sequence length, making it suitable for real-world large-context applications. Furthermore, ablation analysis confirms the critical role of both the external memory module and the BiLSTM controller in achieving improved performance. These findings highlight the effectiveness of explicit memory mechanisms in enhancing both the quality and efficiency of natural language processing systems.

## Data Availability

Publicly available datasets were analyzed in this study. This data can be found here: https://github.com/abisee/cnn-dailymail.
